# Supplementary material for: Ignoring correlated activity causes a failure of retinal population codes
Source: Nat Commun. 2020 Sep 14;11:4605. doi: 10.1038/s41467-020-18436-2 (PMC7490269; doi:10.1038/s41467-020-18436-2)
Supplement: Supplementary file 1 — Supplementary Information [file 41467_2020_18436_MOESM1_ESM.pdf]

Ignoring correlated activity causes a failure of retinal population codes

Supplementary Information

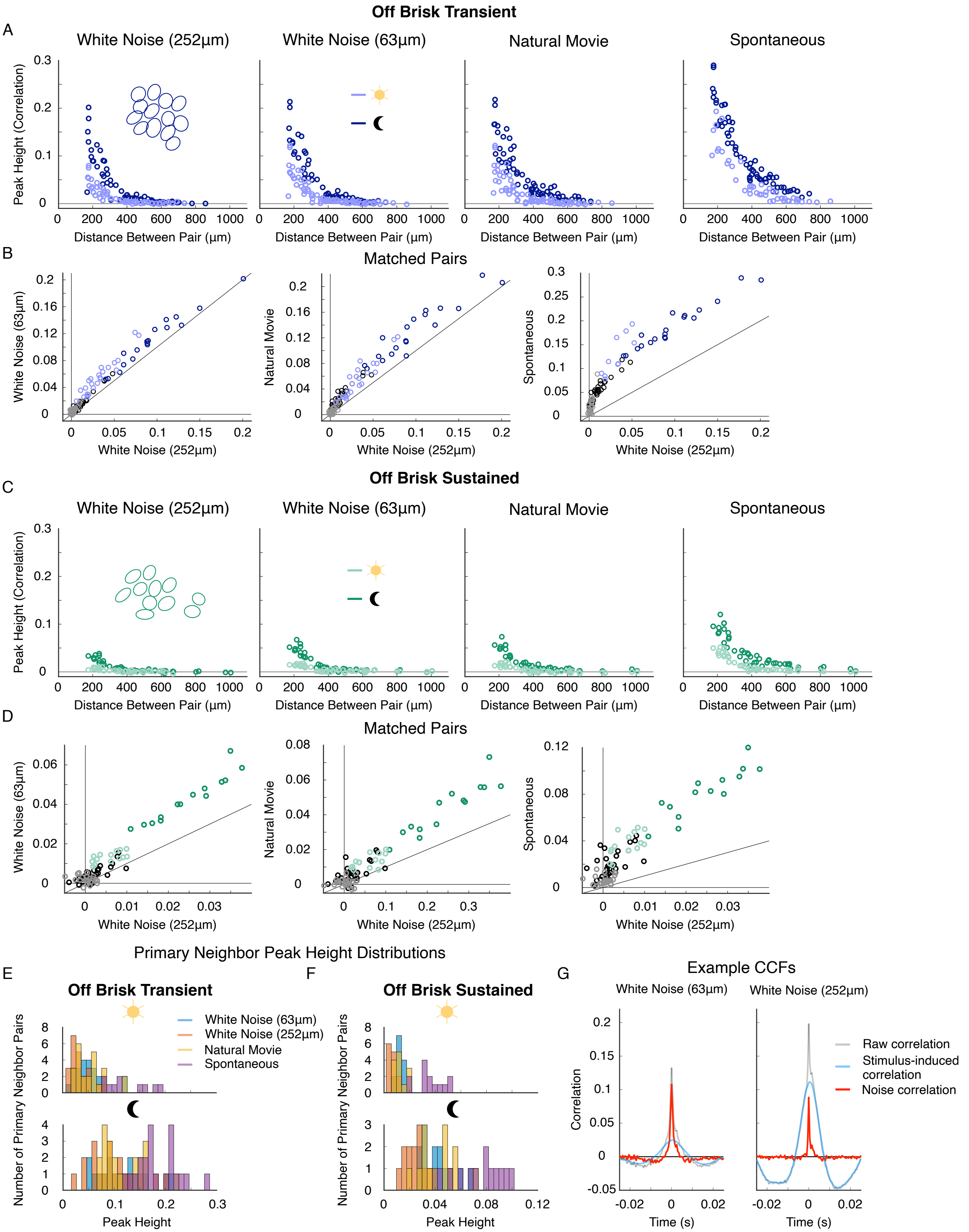

Supplementary Figure 1: Noise correlations depend on light level and cell type no matter the stimulus condition. **A.** Inset: RF mosaic of OFF-bt RGCs. Main panel: Strength of noise correlations for four different stimulus conditions over pairwise distances for the OFF-bt population. Each point shows the cross-correlogram height at 0-time lag for a given pair of cells (78 RGC pairs from 1 retina; photopic light level: 10,000 Rh* rod^{-1} s^{-1}; scotopic light level: 1 Rh* rod^{-1} s^{-1}). Leftmost: Large pixel white noise (252μm squares), which was used for decoding. Second to left: Small pixel white noise (63μm squares). Second to right: A natural movie taken from a camera mounted on a cat's head while walking through a forest [1]. Right: Static, full-screen gray stimulus. **B.** Comparing CCF peak height for the same RGC pairs across different stimuli. Colored points indicate primary neighbor pairs, while gray and black points indicate pairs with larger pairwise distances under the photopic and scotopic light levels, respectively (24 primary neighbor pairs). Left: Large pixel white noise and small pixel white noise. Middle: large pixel white noise and natural stimuli. Right: large pixel white noise and spontaneous activity. **C**-**D.** Same as **A**-**B** for OFF-bs RGCs (45 RGC pairs from the same retina; 13 primary neighbor pairs) **E.** Distribution of CCF heights under each stimulus type for primary neighbor pairs of OFF-bt RGCs (colored points from **B**). **F.** Same as **E.** for OFF-bs RGCs. **G.** Example CCFs of one OFF-bt pair at the scotopic light level showing raw measured correlations (gray), stimulus-induced correlations (shuffle correlogram; blue), and noise correlations (red). Noise correlations are similar for the small pixel white noise (left) and large pixel white noise (right).

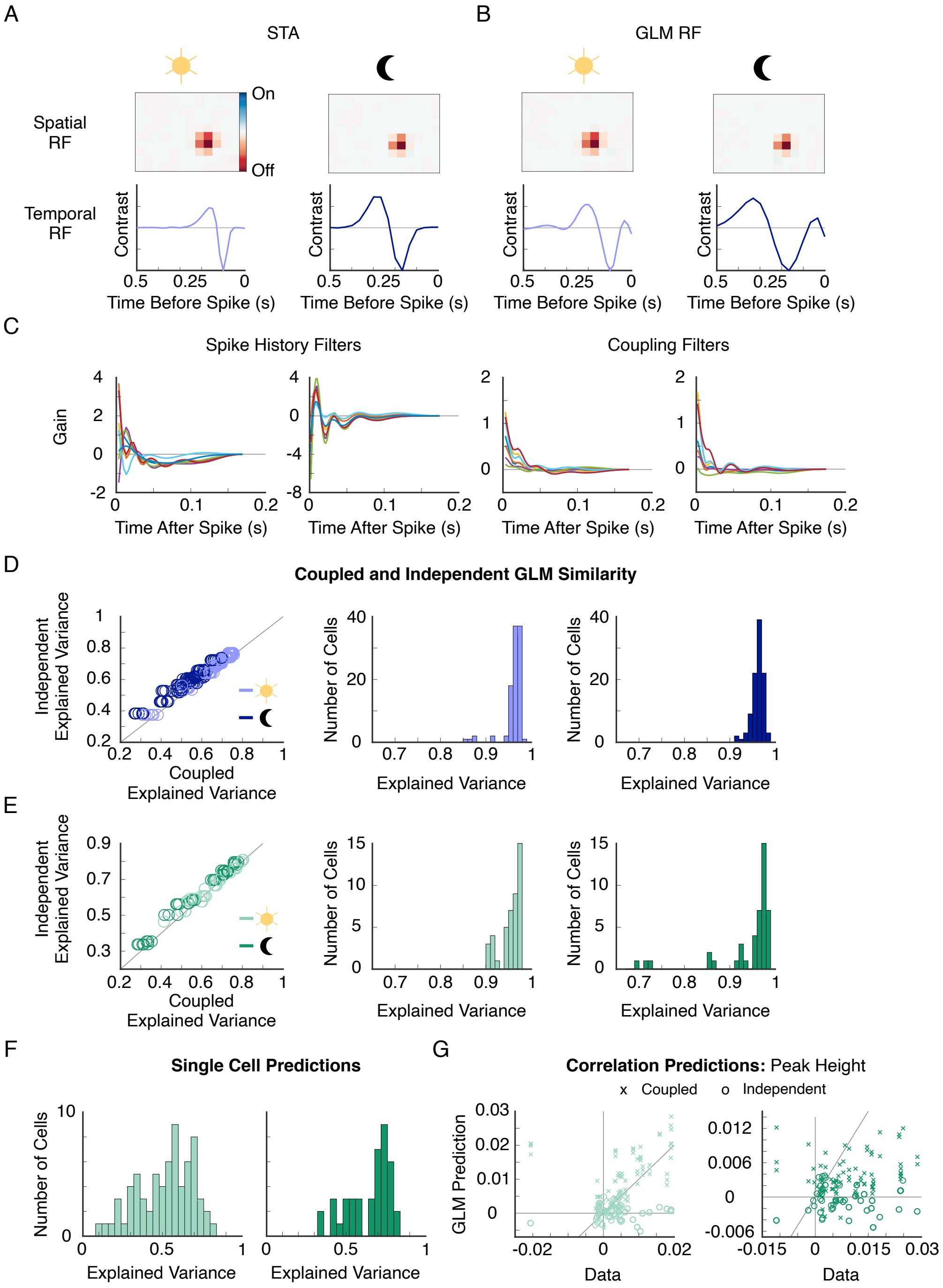

Supplementary Figure 2: Summary of GLM fitting performance. **A.** Spike triggered average (STA) of an example OFF-bt RGC across light levels. Note that these RGCs have space-time separable RFs [2]. Top row, spatial components of the STA estimate the spatial RF. Bottom row, time courses of the STA estimate the temporal RFs (also called temporal filters). Notice the slower time course at the scotopic light level. **B.** Same as **A** but spatial and temporal filters are from the GLM fit. **C.** Example spike history filters (left) and coupling filters (right) at each light level for a coupled group of RGCs. **D.** Comparing coupled and independent GLM performances for OFF-bt RGCs. Left, performances in predicting firing rates are similar for the coupled and independent GLMs (101 cells from 16 groups of RGCs from 1 retina; all data: photopic: independent GLM explained variance = 0.59

± 0.01, mean ± s.e.m., 100 RGCs from 4 retinas, coupled GLM explained variance = 0.59 ± 0.007, 55 groups of RGCs from 4 retinas, scotopic: independent GLM explained variance = 0.58 ± 0.01, 69 RGCs from 3 retinas, coupled GLM explained variance = 0.51 ± 0.008, 37 groups of RGCs from 4 retinas). RGCs were used once in the independent GLMs but many are part of multiple coupled GLMs. Middle, distribution of explained variance between coupled and independent PSTH predictions at the photopic light level (all data: 0.93 ± 0.002). Right, distribution of explained variance between coupled and independent PSTH predictions at the scotopic light level (all data: 0.94 ± 0.002). **E.** Same as **D** for OFF-bs RGCs (44 cells from 8 groups of RGCs from 1 retina; all data: photopic: independent GLM explained variance = 0.52 ± 0.02, 69 RGCs from 4 retinas, coupled GLM explained variance = 0.52 ± 0.01, explained variance between independent and coupled GLMs = 0.9 ± 0.006, 37 groups of RGCs from 4 retinas, scotopic: independent GLM explained variance = 0.65 ± 0.02, 42 RGCs from 3 retinas, coupled GLM explained variance = 0.64 ± 0.01, explained variance between independent and coupled GLMs = 0.94 ± 0.005, 20 groups of RGCs from 3 retinas). **F.** Distribution of explained variances for the GLM predicted PSTHs in OFF-bs RGCs (photopic: 69 RGCs; scotopic: 42 RGCs). **G.** Cross-correlogram peak predictions for the independent and coupled GLMs across the OFF-bs RGC population (left: photopic condition, right: scotopic condition; 102 pairs from 9 groups of RGCs from 1 retina).

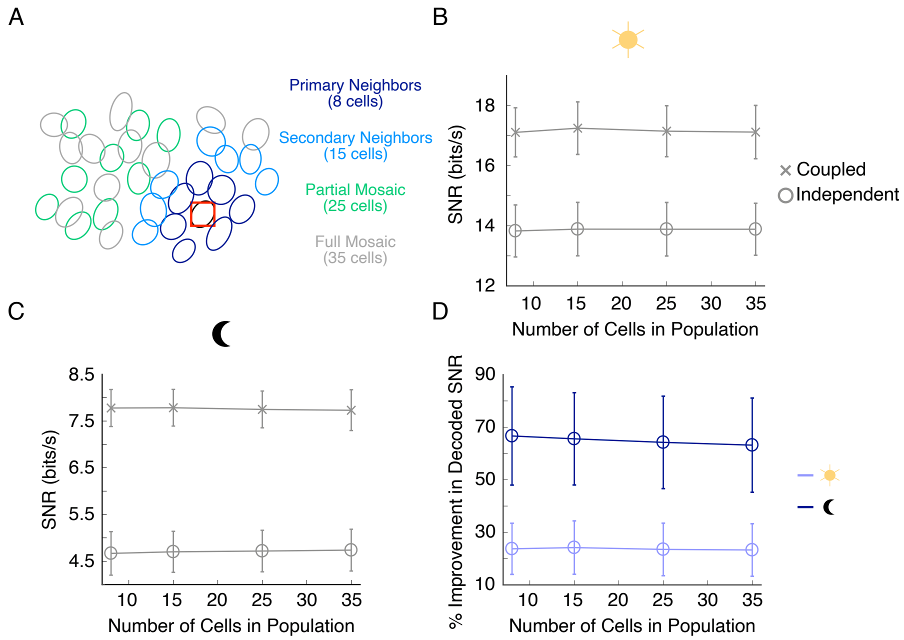

Supplementary Figure 3: Including RGCs beyond immediate neighbors does not significantly impact temporal decoding results. **A.** OFF-bt RF mosaics colored to show the four different populations that were compared with GLM decoding. The first group included an RGC centered over the decoded stimulus pixel and all of its primary neighbors. The second group adds secondary neighbors, the third group adds some far away RGCs, and the fourth group uses the whole recorded population. **B.** Decoded SNR for the independent and coupled GLMs fit with the groups in **A** at the photopic light level. **C.** Same as **B** for the scotopic light level. **D.** Percent improvement in decoded SNR between the coupled and independent GLMs for the different RGC groups. All error bars (s.d.) come from bootstrapping SNR.

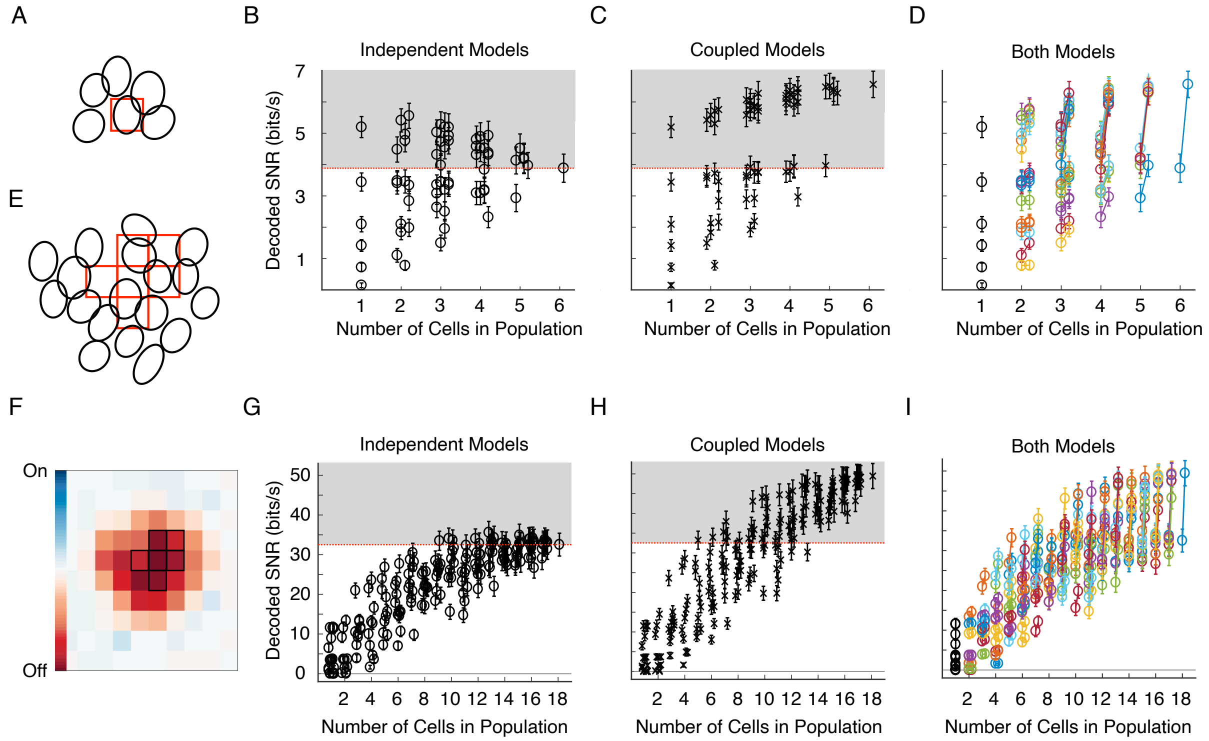

Supplementary Figure 4: Population failure as a function of group size for decoding in time (panels **A**-**D**) or space (panels **E**-**I**). **A.** RF mosaic for 6 OFF-bt RGCs used to decode the temporal sequence of the stimulus pixel in red. **B.** Decoded SNR for independent GLMs as a function of number of cells included in the population. All possible combinations of 1-6 RGCs were used. The independent GLM with all 6 cells decodes less information than GLMs with some combinations of 1-6 RGCs. Points are slightly jittered in the x direction for visualization. **C.** Same as **B** for coupled GLMs. Several combinations of 2-6 coupled GLMs (including 1 single cell GLM) decode more information than the independent GLM with 6 cells (gray region). **D.** Decoded SNR for independent and coupled GLMs, where models using the same group of RGCs are connected by a line. For visualization, the coupled SNRs are plotted slightly to the right of the independent SNRs. **E.** RF mosaic for 18 OFF-bt RGCs used to decode the spatial pattern of stimulus pixels in red. Here we consider when an independent GLM consisting of many RGCs decodes worse than a GLM made up of a smaller population. This scenario exhibits population failure because the decoder with more cells fails to take advantage of the information provided by larger population input. Expanding to larger populations in this way is necessary because of the large size of stimulus pixels relative the RGC RFs, which makes it unlikely that a single RGC can decode a large spatial pattern well. **F.** Cumulative RF coverage for the 18 RGCs. The spatial pattern of stimulus pixels that was decoded is outlined in black. This plot shows that the decoded stimulus pixels are well represented by the group of RGCs. **G.** Decoded SNR for independent GLMs as a function of number of cells included in the population. For groups with 2-17 RGCs, all possible combinations of RGCs were subsampled. Note that decoded SNR appears to plateau at ~15 cells. **H.** Same as **G** for coupled GLMs. The independent GLM using all 18 RGCs decodes less information than coupled GLMs with some combinations of 7, 9-17 RGCs (gray region). Note that decoded SNR would likely continue

growing with a larger population of RGCs. **I.** Decoded SNR for independent and coupled GLMs, where models using the same group of RGCs are connected by a line. For visualization, the coupled SNRs are plotted slightly to the right of the independent SNRs. For a population of 18 RGCs, the coupled model performs 52 $\pm$ 16.61 % (mean $\pm$ s.d.) better than the independent model. All error bars are s.d. from bootstrapping.

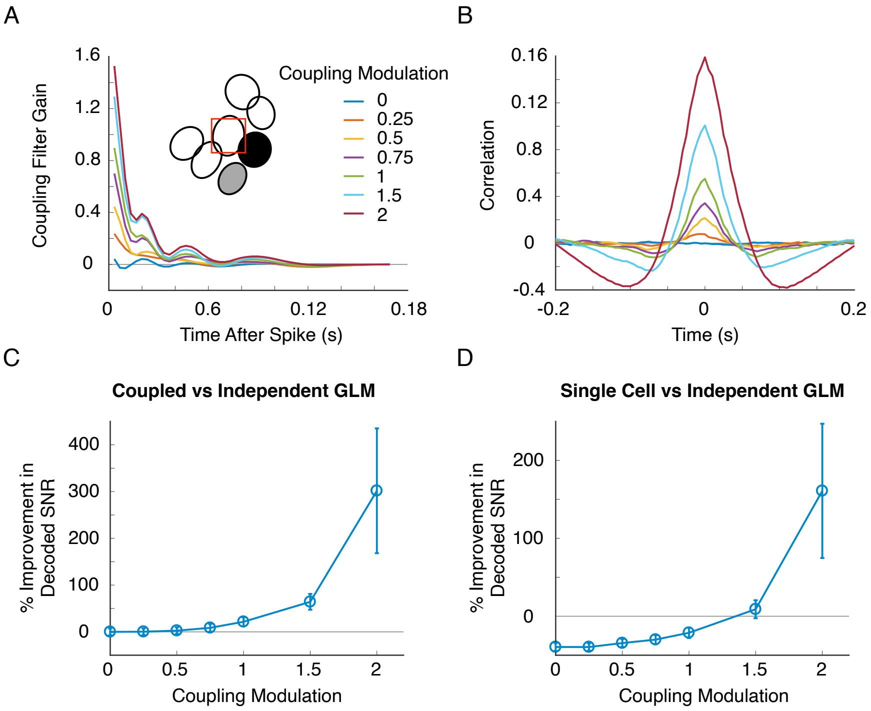

Supplementary Figure 5: Stronger coupling causes a greater improvement in decoding when accounting for correlations relative to assuming independence. We started with a coupled GLM fit to a population of OFF-bt RGCs at the scotopic light level (inset in **A**). We then altered all coupling filters in the model by a constant factor, simulated spike trains, refit coupled and independent GLMs, and decoded stimuli from the simulated spike trains to determine the impact of directly altering coupling strengths on decoding performance. **A.** Coupling filters between two RGCs in the population (highlighted in the inset) for the indicated coupling modulation factors. These coupling filters were used in the GLM to simulate responses. **B.** Resulting noise CCFs from simulated spike trains of the gray and black cells in **A**. **C.** Percent improvement in decoded SNR between the coupled and independent GLM as a function of coupling modulation. Stronger coupling filters make ignoring correlated activity more deleterious for decoding. **D.** Same as **C** but comparing decoding with a single cell and the independent GLM to demonstrate population failure. Population failure does not occur for the original group of cells (coupling modulation = 1), but increasing coupling strength causes the independent GLM to decode less information than the single cell. All error bars are s.d. from bootstrapping.

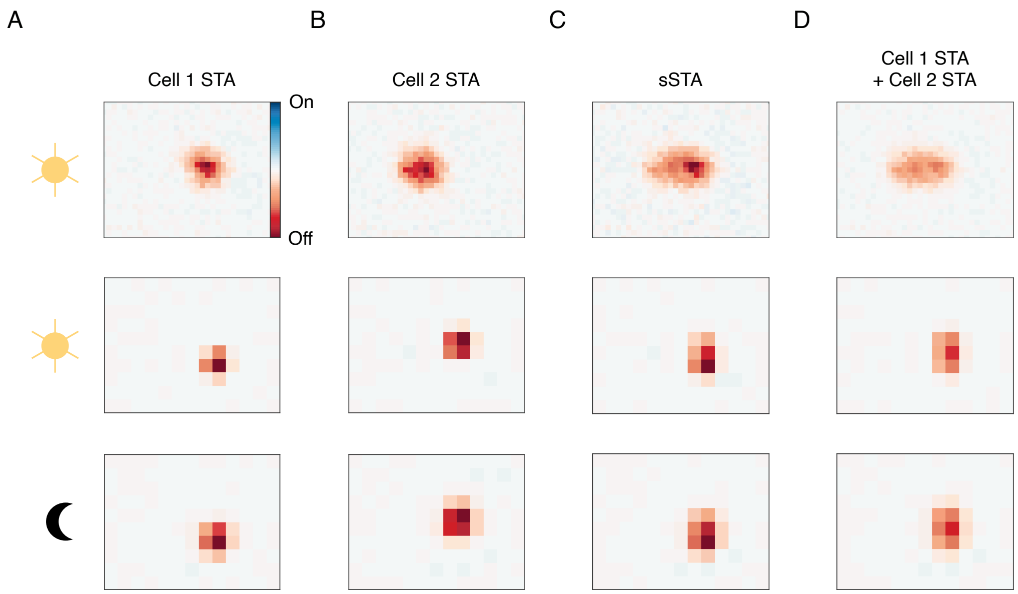

Supplementary Figure 6: Synchronous spikes between pairs of RGCs do not encode finer spatial information. **A.** Top, spatial RF of an example OFF-bt RGC at the photopic light level measured with a fine spatial resolution. Middle, the same cell's RF at the spatial resolution used for GLM decoding at the photopic light level. Bottom, the cell's RF at the scotopic light level. **B.** RFs for an RGC neighboring the cell in **A**. **C.** Synchronous spike triggered averages (sSTAs) between the RGCs of **A** & **B**. The sSTAs do not resemble the intersection of the two individual RFs. **D.** The union of the RFs from **A** & **B**.

Supplementary References

1 Betsch, B., Einhauser, W., Kording, K. & Konig, P. The world from a cat's perspective--statistics of natural videos. *Biol Cybern* **90**, 41-50 (2004).

2 Ravi, S., Ahn, D., Greschner, M., Chichilnisky, E. J. & Field, G. D. Pathway-Specific Asymmetries between ON and OFF Visual Signals. *J Neurosci* **38**, 9728-9740, doi:10.1523/JNEUROSCI.2008-18.2018 (2018).
