# Supplementary material for: Ignoring correlated activity causes a failure of retinal population codes
Source: Nat Commun. 2020 Sep 14;11:4605. doi: 10.1038/s41467-020-18436-2 (PMC7490269; doi:10.1038/s41467-020-18436-2)
Supplement: Supplementary file 3 — Reporting Summary [file 41467_2020_18436_MOESM3_ESM.pdf]

nature research

Corresponding author(s): Greg D Field

Last updated by author(s): 07/10/2020

Reporting Summary

Nature Research wishes to improve the reproducibility of the work that we publish. This form provides structure for consistency and transparency in reporting. For further information on Nature Research policies, see Authors & Referees and the Editorial Policy Checklist.

Statistics

For all statistical analyses, confirm that the following items are present in the figure legend, table legend, main text, or Methods section.

n/a	Confirmed	
☐	☒	The exact sample size (*n*) for each experimental group/condition, given as a discrete number and unit of measurement
☐	☒	A statement on whether measurements were taken from distinct samples or whether the same sample was measured repeatedly
☐	☒	The statistical test(s) used AND whether they are one- or two-sided<br>*Only common tests should be described solely by name; describe more complex techniques in the Methods section.*
☐	☒	A description of all covariates tested
☐	☒	A description of any assumptions or corrections, such as tests of normality and adjustment for multiple comparisons
☐	☒	A full description of the statistical parameters including central tendency (e.g. means) or other basic estimates (e.g. regression coefficient) AND variation (e.g. standard deviation) or associated estimates of uncertainty (e.g. confidence intervals)
☐	☒	For null hypothesis testing, the test statistic (e.g. *F*, *t*, *r*) with confidence intervals, effect sizes, degrees of freedom and *P* value noted<br>*Give P values as exact values whenever suitable.*
☒	☐	For Bayesian analysis, information on the choice of priors and Markov chain Monte Carlo settings
☒	☐	For hierarchical and complex designs, identification of the appropriate level for tests and full reporting of outcomes
☐	☒	Estimates of effect sizes (e.g. Cohen's *d*, Pearson's *r*), indicating how they were calculated

Our web collection on statistics for biologists contains articles on many of the points above.

Software and code

Policy information about availability of computer code

Data collection	Visual stimuli were displayed using publicly available MGL codebase available at: http://gru.stanford.edu/doku.php/mgl/overview. Multielectrode array voltages were acquired with customized Labview software. This acquisition code will be made available upon reasonable request to the corresponding author.
Data analysis	Spike sorting was performed with custom Java software. Most data analysis was performed with open source code previously published and distribute by Dr. Jonathan Pillow at Princeton University (https://github.com/pillowlab/GLMspiketools). Other modeling code is provided at https://github.com/kmruda/RGC-population-failure.

For manuscripts utilizing custom algorithms or software that are central to the research but not yet described in published literature, software must be made available to editors/reviewers. We strongly encourage code deposition in a community repository (e.g. GitHub). See the Nature Research guidelines for submitting code & software for further information.

Data

Policy information about availability of data

All manuscripts must include a data availability statement. This statement should provide the following information, where applicable:

- Accession codes, unique identifiers, or web links for publicly available datasets
- A list of figures that have associated raw data
- A description of any restrictions on data availability

The data from an example recording generated and analyzed in this study are available at https://doi.org/10.12751/g-node.cnqoty. The full dataset that supports the findings of this study are available from the corresponding author upon reasonable request.

Field-specific reporting

Please select the one below that is the best fit for your research. If you are not sure, read the appropriate sections before making your selection.

☒ Life sciences ☐ Behavioural & social sciences ☐ Ecological, evolutionary & environmental sciences

For a reference copy of the document with all sections, see nature.com/documents/nr-reporting-summary-flat.pdf

Life sciences study design

All studies must disclose on these points even when the disclosure is negative.

Sample size	Four retinas with 20-55 groups of retinal ganglion cells (depending on cell type and light level) was determined to be sufficient because all retinas exhibited the same trends.
Data exclusions	One retina (from one animal) was excluded because the visual stimulus was not properly focused onto the photoreceptors.
Replication	All attempts at replication were successful.
Randomization	Randomization is not relevant to this study because the goal was to compare across different retinal ganglion cell types and light levels.
Blinding	Blinding is not relevant to this study because the results (noise correlation values and decoding performance across light levels and cell types) do not depend on decisions that could be biased by the investigators.

Reporting for specific materials, systems and methods

We require information from authors about some types of materials, experimental systems and methods used in many studies. Here, indicate whether each material, system or method listed is relevant to your study. If you are not sure if a list item applies to your research, read the appropriate section before selecting a response.

Materials & experimental systems

n/a	Involved in the study	
☒	☐	Antibodies
☒	☐	Eukaryotic cell lines
☒	☐	Palaeontology
☐	☒	Animals and other organisms
☒	☐	Human research participants
☒	☐	Clinical data

Methods

n/a	Involved in the study	
☒	☐	ChIP-seq
☒	☐	Flow cytometry
☒	☐	MRI-based neuroimaging

Animals and other organisms

Policy information about studies involving animals; ARRIVE guidelines recommended for reporting animal research

Laboratory animals	Long-Evans rats, females, age 51-215 days
Wild animals	No wild animals were collected
Field-collected samples	No field-collected samples were used
Ethics oversight	Institutional Animal Care and Use Committee at Duke University

Note that full information on the approval of the study protocol must also be provided in the manuscript.
